# In Silico Studies and Biological Evaluation of Thiosemicarbazones as Cruzain-Targeting Trypanocidal Agents for Chagas Disease

**DOI:** 10.3390/pharmaceutics18010065

**Published:** 2026-01-04

**Authors:** Lidiane Meier, Milena F. C. V. de Melo, Heitor R. Abreu, Isabella M. e Oliveira, Larissa Sens, Thiago H. Doring, Renata Krogh, Adilson Beatriz, Adriano D. Andricopulo, Sumbal Saba, Aldo S. de Oliveira, Jamal Rafique

**Affiliations:** 1 Department of Exact Sciences and Education, Blumenau Campus, Federal University of Santa Catarina, Blumenau 89065-100, SC, Brazil; 2 Instituto de Química, Universidade Federal do Mato Grosso do Sul (UFMS), Campo Grande 79074-460, MS, Brazil; 3 LabSO, Instituto de Química, Universidade Federal de Goiás (UFG), Goiânia 74690-900, GO, Brazilsumbalsaba@ufg.br (S.S.); 4 Instituto Federal do Espírito Santo, Campus Itapina, Colatina 29717-000, ES, Brazil; 5 Laboratory of Medicinal and Computational Chemistry (LQMC), São Carlos Institute of Physics (IFSC), University of Sao Paulo (USP), São Carlos 13563-120, SP, Brazil; 6 Gulbenkian Institute for Molecular Medicine (GIMM), Faculty of Medicine, University of Lisbon, 1649-028 Lisbon, Portugal

**Keywords:** ADMET, Chagas disease, cruzain inhibition, molecular docking, thiosemicarbazones

## Abstract

**Background/Objectives:** Chagas disease remains a major unmet medical need due to the limited efficacy and safety of current therapies. Here, we investigated sixteen thiosemicarbazone (TSC) derivatives as cruzain inhibitors using an integrated in silico/in vitro workflow. Methods: Docking against cruzain (PDB 3KKU) guided hit prioritization and correlated with enzyme inhibition; validation by redocking supported the protocol’s reliability. **Results**: The top compounds—H7, H10 and H11—showed potent cruzain inhibition (IC_50_ = 0.306, 0.512 and 0.412 µM, respectively) and low-micromolar trypanocidal activity, with negligible cytotoxicity in human fibroblasts (CC_50_ > 64 µM) and favorable selectivity. Structure–activity insights highlighted the role of expanded aromatic systems and electron-donating groups in enhancing binding within S2/S1′ subsites, while nitro substituents were associated with higher cytotoxicity. In silico ADMET parameters supported oral drug-likeness and acceptable metabolic liabilities. **Conclusions**: Overall, these data position TSCs as promising anti-T. cruzi leads and underscore the value of rational design against cruzain.

## 1. Introduction

Chagas disease (CD), a debilitating illness caused by the protozoan parasite Trypanosoma cruzi (T. cruzi)and remains a significant public health concern, particularly across Latin America. As a neglected tropical disease, it afflicts millions and often leads to severe, irreversible cardiac and gastrointestinal complications over decades of chronic infection [1,2]. The therapeutic arsenal against CD is limited to two nitroheterocyclic drugs, benznidazole and nifurtimox. Their use is hampered by frequent severe adverse effects and limited efficacy, particularly in the chronic phase of the disease, which often leads to high rates of treatment discontinuation and therapeutic failure [3,4]. Thus, novel therapeutic strategies are urgently required to address this unmet medical need.

Cruzain, the major cysteine protease of *T. cruzi*, is a validated drug target due to its parasite survival, differentiation, and virulence [5,6]. Consequently, structure-based drug design and high-throughput screening have led to the identification of several cruzain inhibitors with promising preclinical efficacy [7,8].

In this regard, thiosemicarbazones (TSCs) have shown significant biological activities, including antiparasitic properties, and have emerged as a promising scaffold for the development of new cruzain inhibitors [9,10]. Recent studies show that rationally designed TSCs exhibit inhibitory activity against cruzain ranging from micromolar to nanomolar levels, often with improved selectivity and pharmacokinetic properties compared to existing drugs [11,12]. The versatility of the TSC scaffold enables extensive chemical modifications, facilitating the optimization of pharmacodynamic and pharmacokinetic properties [13].

As part of our wider research interest in bioactive compounds [14,15,16,17,18,19,20,21,22], herein we present the studies of in silico molecular modeling, enzymatic inhibition assays, phenotypic trypanocidal testing, cytotoxicity evaluation, and ADMET predictions to systematically investigate a series of sixteen TSC derivatives as cruzain inhibitors. By integrating computational and experimental approaches, we aim to identify promising candidates for further preclinical development and to provide new insights into the structure–activity relationships governing cruzain inhibition and anti-*T. cruzi* activity.

We hypothesized that TSC derivatives bearing expanded aromatic surfaces and electron-donating groups would interact optimally with the cruzain S2/S1′ subsites, resulting in higher potency and selectivity. Accordingly, our objective was to integrate molecular modeling, enzymatic assays, and phenotypic testing to identify and characterize promising TSC candidates against *T. cruzi*.

## 2. Materials and Methods

All reagents were purchased from commercial sources (Sigma-Aldrich, São Paulo, Brazil), and all solvents used were of analytical grade, without further purification.

### 2.1. Chemistry

All sixteen thiosemicarbazone (TSC) derivatives were previously synthesized and thoroughly characterized (^1^H-NMR, ^13^C-NMR, and HRMS) as reported by us [23]. The compounds utilized in the present biological investigations are from these same authenticated batches. In general, the synthesis involved a two-step procedure. The synthesis began with the reaction of 4-methoxyphenyl isothiocyanate (10 mmol) and hydrazine hydrate (50%, 20 mmol) in isopropanol (20 mL) at ambient temperature for 3 h, which precipitated the key thiosemicarbazide intermediate. This intermediate was isolated by vacuum filtration (Prismatec vaccum pump, model 121, max. displacement 2.2 m^3^/h–Prismatec, ITU/SP, Brazil), thoroughly washed with isopropanol, and then used directly in the next step. Subsequently, this thiosemicarbazide (1.19 mmol) was condensed with a diverse set of aldehydes (1.25 mmol) in a refluxing ethanol/water solution (11 mL/22 mL) catalyzed by acetic acid. After one hour, the mixture was cooled to room temperature, precipitating the final TSC product, which was collected by vacuum filtration and washed with water.

### 2.2. In Silico Studies

A comprehensive in silico workflow [24] was employed to design, optimize, and evaluate the interactions between sixteen TSCs derivatives and cruzain, the major cysteine protease of *T. cruzi*.

Initially, the two- and three-dimensional structures of all TSCs derivatives, were constructed using ACD/ChemSketch^®^ v2021.1 (Advanced Chemistry Development, Toronto, ON, Canada). The geometries were further optimized with Avogadro^®^ v1.2.0 (open-source molecular builder, Pittsburgh, PA, USA, https://avogadro.cc (accessed 2 August 2025) to ensure suitability for molecular docking analyses.

The high-resolution crystal structure of cruzain (PDB ID: 3KKU, 1.28 Å) [25], obtained from the Protein Data Bank (PDB), was selected as the biological target. The co-crystallized ligand was used to define the active site, and all water molecules were removed to reduce computational complexity, except for those located within the catalytic site and known to be essential for enzymatic activity.

Molecular docking simulations were conducted using GOLD^®^ v.2022.1 [26,27], using 20 GA runs per ligand, population size of 100, and search efficiency of 100% applying four scoring functions (ASP, ChemScore, GoldScore, and ChemPLP) to comprehensively assess ligand–enzyme interactions. Ligands were treated as flexible, while the protein structure was kept rigid. The docking search space was defined as a sphere with a 6 Å radius centered on the geometric center of the co-crystallized ligand. Docking protocol validation was performed via re-docking, with root-mean-square deviation (RMSD) values calculated to confirm the reliability of the predicted binding poses.

Docking results were analyzed and visualized using BIOVIA Discovery Studio Visualizer v2024 (Dassault Systèmes, San Diego, CA, USA) and PyMOL^®^ v2.5.5 (Schrödinger LLC, New York City, NY, USA). In silico predictions of pharmacokinetic and toxicological properties for all TSC derivatives, as well as the reference drugs benznidazole and nifurtimox, were carried out using Molinspiration^®^ (http://www.molinspiration.com; accessed 2 August 2025, Slovensky Grob, Slovak Republic), OSIRIS Property Explorer (https://www.organic-chemistry.org/prog/peo/; accessed 2 August 2025, Allschwil, Switzerland), SwissADME (http://www.swissadme.ch; accessed 2 August 2025, Lausanne, Switzerland), and pkCSM (http://biosig.unimelb.edu.au/pkcsm; accessed 2 August 2025, Melbourne, Australia) [28]. Correlations between computational and experimental data were evaluated through linear regression analyses using GraphPad Prism v9.5.1 (GraphPad Software, San Diego, CA, USA).

### 2.3. In Vitro Assays Against Cruzain

The catalytic activity of cruzain was assessed using the fluorogenic substrate benzyloxycarbonyl-phenylalanine-arginine-7-amido-4-methylcoumarin (Z-Phe-Arg-AMC), which releases 7-amino-4-methylcoumarin upon cleavage, allowing fluorescence-based quantification. Recombinant cruzain (≥90% purity) was obtained from BPS Bioscience (Cat. No. 100101, San Diego, CA, USA) and handled according to the supplier’s protocol. Excitation and emission wavelengths were set at 355 nm and 460 nm, respectively. Enzymatic assays were performed in 96-well plates under the following conditions: 1.0 nmol L^−1^ cruzain, 5.0 μmol L^−1^ Z-Phe-Arg-AMC substrate, 5 mmol L^−1^ DTT, 0.01% Triton X-100, and sodium acetate buffer (0.1 mol L^−1^, pH 5.5). Fluorescence was monitored for 300 s at 30 °C. Results were expressed as IC_50_ values [8].

### 2.4. In Vitro Assays Against Trypanosoma Cruzi

In vitro activity against *T. cruzi* was evaluated using Tulahuen lacZ strains (kindly provided by collaborators and is available through COLPROT/FIOCRUZ), genetically modified to express the *Escherichia coli* β-galactosidase (lacZ) gene. The substrate chlorophenol red β-D-galactopyranoside (CPRG, Sigma Chemical Co., St. Louis, MO, USA) was used for colorimetric detection. Epimastigote forms of *T. cruzi* were cultured in liver infusion tryptose (LIT) medium supplemented with 10% fetal bovine serum (FBS), penicillin, and streptomycin. Trypomastigotes were collected from the supernatant of infected cell cultures. Human fibroblasts HFF-1 cells (ATCC^®^ 77CRL-1635™) (2.0 × 10^3^ cells/well) were seeded in 96-well plates containing 80 μL of Roswell Park Memorial Institute Medium (RPMI) 1640 (without phenol red) supplemented with 10% FBS and antibiotics and incubated overnight. Subsequently, 1.0 × 10^4^ trypomastigotes were added per well in 20 μL of medium. After 24 h, TSC derivatives were added in serial dilutions (50 μL, 100–0.1 μM range) in duplicate. After 72 h of incubation, cultures were microscopically inspected for sterility and parasite growth. Then, parasite viability was quantified by absorbance measurement at 570 nm using a SpectraMax^®^ 190 microplate reader (Molecular Devices, San Jose, CA, USA) [29].

### 2.5. Cytotoxicity Assays in Human Fibroblasts (HFF1)

The cytotoxicity of the compounds was evaluated by the tetrazolium salt (MTS) assay on human foreskin fibroblast HFF1 cells [30]. The HFF-1 cells (ATCC^®^ CRL-1635™) were obtained directly from ATCC and maintained in RPMI 1640 supplemented with 10% fetal bovine serum and antibiotics under 5% CO_2_ at 37 °C. Cells were seeded at a density of 2.0 × 10^3^ cells per well in 96-well plates and incubated overnight. Test compounds were added in seven concentrations (100–0.1 µM) in triplicate. After 72 h of incubation at 37 °C in a 5% CO_2_ humidified atmosphere, 20 μL of MTS reagent (CellTiter 96^®^ AQueous One Solution Cell Proliferation Assay, Promega, Madison, WI, USA) were added to each well, followed by further incubation for 4 h. Absorbance at 490 nm was measured to evaluate viable cells. The percentage of nonviable cells was calculated relative to negative control wells (0.5% dimethylsulfoxide, DMSO). Each experiment was independently repeated at least twice.

## 3. Results and Discussion

**Figure 1:**
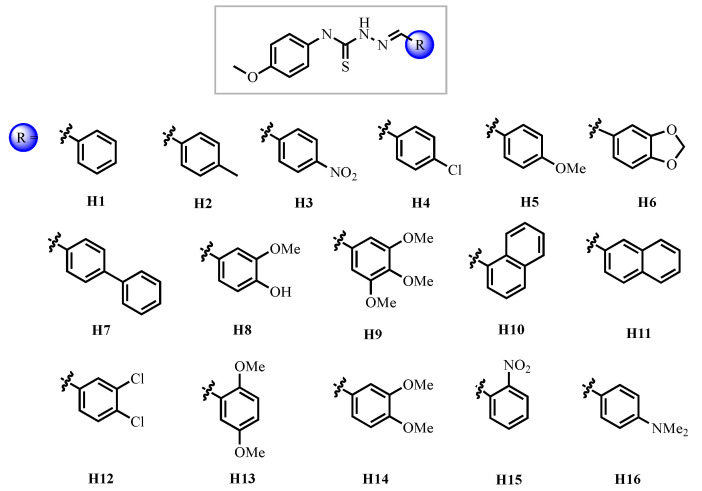
Series of synthesized TSCs evaluated in this work.

This comprehensive study evaluated 16 TSC derivatives (Figure 1) through a multidisciplinary approach involving in silico and in vitro enzymatic inhibition assays against cruzain, phenotypic trypanocidal activity tests, cytotoxicity assays on mammalian cells, pharmacokinetic and pharmacodynamic predictions. The central biological target was cruzain, a cysteine protease critical for *T. cruzi* survival, differentiation, and host cell invasion.

### 3.1. Trypanocidal Activity and Cytotoxicity

In vitro assays against bloodstream trypomastigotes of *T. cruzi* confirmed the potent antiparasitic activity of **H7**, **H10**, and **H11**, with CC_50_ values of 1.96, 2.85, and 2.15 µM, respectively (Table 1). The alignment between enzymatic inhibition and trypanocidal potency supports the hypothesis that cruzain inhibition is a primary mechanism of action.

The phenotypic assays further revealed that the compounds with the most potent cruzain inhibitory profiles consistently translated this efficacy into substantial antiparasitic activity, highlighting a direct functional link between enzymatic inhibition and parasite death. The congruence between these two experimental arms suggests that the compounds disrupt critical proteolytic processes essential for parasite viability, notably impairing the ability of *T. cruzi* to invade host cells and maintain intracellular replication. Additionally, the disruption of cruzain activity likely compromises the parasite’s evasion mechanisms against the host immune response, further contributing to parasite clearance.

In addition to the strong correlation observed between docking scores (ChemPLP) and cruzain enzymatic inhibition (R^2^ = 0.9805), a moderate yet significant correlation was also found between docking predictions and phenotypic assays against *T. cruzi* (R^2^ = 0.6418). This finding reinforces the role of cruzain as a biologically relevant target and supports the hypothesis that the trypanocidal activity of the tested compounds is, at least in part, mediated by cruzain inhibition.

The statistical analysis suggests that while cellular activity involves additional biological complexities, molecular docking remains a valuable predictive tool, particularly for early-stage compound screening. The intermediate correlation implies that the top-performing compounds structurally—such as **H7**, **H10**, and **H11**—not only display strong enzyme binding affinity but also maintain efficacy in a more complex biological context. These results highlight the strategic utility of virtual screening in prioritizing lead compounds with both strong target engagement and phenotypic effectiveness.

Cytotoxicity evaluations using human fibroblast cells (HFF-1) revealed that most TSCs exhibited minimal toxicity, with selectivity indices (SI) favoring antiparasitic activity over mammalian cytotoxicity. This favorable safety profile is particularly important for potential therapeutic applications, as it indicates a window of selectivity that could minimize side effects in future in vivo studies. The low cytotoxicity observed for **H7**, **H10**, and **H11** suggests that these compounds selectively target parasitic cells without inducing significant damage to human fibroblasts, an essential requirement for advancing these candidates to further preclinical testing.

However, exceptions were noted for compounds **H3** and **H15**, both of which contained nitro groups. These chemical features are often associated with increased oxidative stress, DNA damage, and genotoxicity, which likely contributed to the elevated cytotoxicity observed. This highlights the need for cautious structural modifications, particularly avoiding functionalities that may predispose compounds to off-target toxicity.

Overall, the data demonstrate that the structural attributes optimizing cruzain inhibition not only enhance antiparasitic potency but also largely preserve mammalian cell viability. This dual advantage underscores the therapeutic potential of carefully designed TSC derivatives and provides a solid foundation for their further development as anti-Chagas drug candidates.

### 3.2. Molecular Docking and Enzymatic Inhibition

**Figure 2:**
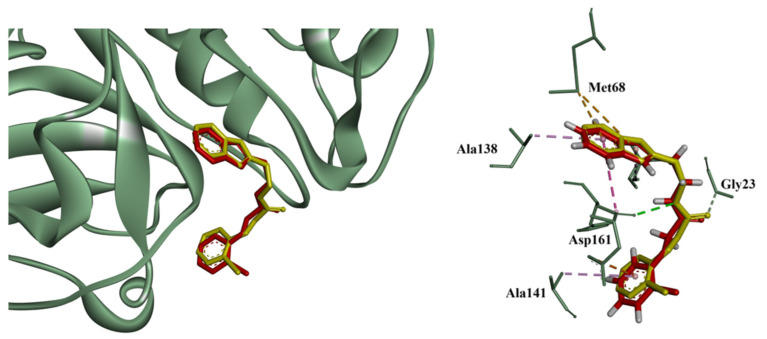
Redocking of the co-crystallized ligand N-(1-benzyl-1H-imidazol-2-yl)-1H-benzimidazol-2-amine into the cruzain active site (PDB ID: 3KKU). The experimental pose is shown in yellow, while the best redocked pose obtained using the ChemPLP scoring function is shown in red. Key interacting residues (Met68, Gly23, Ala138, Ala141, Asp161) are labeled, and hydrogen atoms are explicitly displayed in the ligand. Hydrogen bonds are represented as purple dashed lines, while π–sulfur and π–alkyl interactions are represented as brown dashed lines. The overlap between the two conformations (RMSD = 0.25 Å) confirms the reliability of the docking protocol. Image generated with BIOVIA Discovery Studio Visualizer v24.1.0.23298 [31].

Docking protocol validation was performed through a redocking study of the co-crystallized ligand in the cruzain structure (PDB ID: 3KKU). All four scoring functions available in GOLD^®^—ASP, ChemScore, GoldScore, and ChemPLP—were tested. Among these, ChemPLP achieved the lowest root-mean-square deviation (RMSD) between the redocked and experimental pose, with an RMSD value of 0.25 Å, indicating excellent agreement with the crystallographic conformation and confirming the reliability of the docking protocol (Figure 2).

Subsequent docking simulations using the ChemPLP scoring function revealed that all TSC derivatives exhibited favorable interactions with cruzain (PDB ID 3KKU), with docking scores correlating significantly with the experimental IC_50_ values (R^2^ = 0.9805).

**Figure 3:**
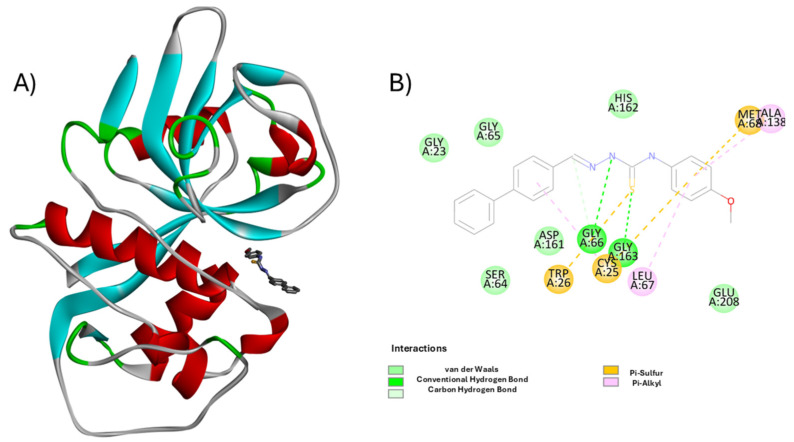
Molecular interaction profile of compound **H7** within the cruzain active site. (**A**) 3D representation of cruzain (PDB ID: 3KKU) with compound **H7** bound at the catalytic site. The protein is shown as a ribbon cartoon (α-helices in red, β-sheets in cyan, loops in gray/green), and the ligand is rendered as sticks in dark gray. (**B**) 2D schematic of ligand–residue interactions highlighting key contacts: van der Waals (green), conventional hydrogen bonds (dark green), carbon hydrogen bonds (light green), π–sulfur (orange), and π–alkyl interactions (purple). Catalytic residue **Cys25** and key stabilizing residues such as **Gly66**, **Asp161**, **Met68**, and **Leu67** are prominently involved. Image generated using BIOVIA Discovery Studio Visualizer v24.1.0.23298 [30].

Molecular visualization of the cruzain—**H7** complex (Figure 3) confirms a highly favorable binding pose within the enzyme’s active site. The ligand establishes a dense interaction network with key residues involved in substrate recognition and stabilization.

Notably, **H7** forms conventional hydrogen bonds with **Gly66**, **Asp161**, and **Gly163**, anchoring the central scaffold of the molecule deep within the catalytic cleft. Additional carbon hydrogen bonding with **Cys25**, along with van der Waals contacts involving **Gly23**, **Ser64**, and **Gly65**, further stabilizes the binding conformation. Hydrophobic interactions also play a crucial role. A *π–sulfur* interaction is observed with **Met68**, while *π–alkyl* interactions are established with **Leu67** and **Ala138**, particularly at the ligand’s terminal aromatic ring. These contacts suggest effective accommodation of this hydrophobic moiety within the S2 subsite of cruzain—a region characterized by its lipophilic environment.

Importantly, **Cys25**, the catalytic residue, is positioned in close proximity to the thiosemicarbazone moiety, supporting the proposed inhibition mechanism via nucleophilic attack on the thiocarbonyl group. This spatial alignment indicates potential for covalent reversible inhibition. Together, the combination of polar and hydrophobic interactions explains the high inhibitory potency observed for **H7** (IC_50_ = 0.306 µM). The compound’s topology enables deep, directed insertion into the active site, maximizing contacts with critical residues. These structural insights highlight **H7** as a chemically and sterically optimized cruzain inhibitor within the TSC series.

**Figure 4:**
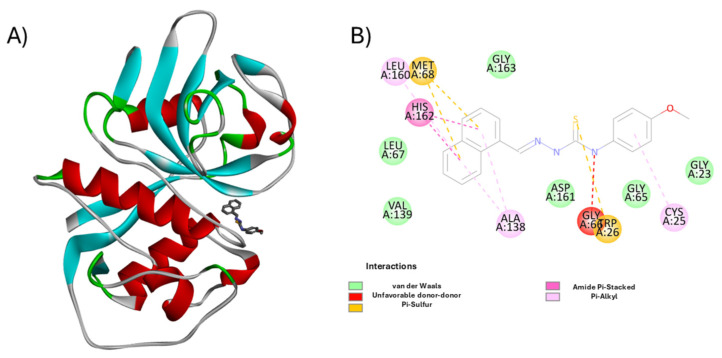
Molecular interaction profile of compound **H10** in the cruzain active site. (**A**) 3D representation of cruzain (PDB ID: 3KKU) showing the ligand **H10** (sticks in dark gray) bound at the catalytic site. Secondary structure elements are shown in cartoon format (α-helices in red, β-sheets in cyan, loops in gray/green). (**B**) 2D schematic of ligand–residue interactions. Notable contacts include π–sulfur interactions (orange) with **Met68** and **Trp26**, π–alkyl interactions (pink) with **Leu160**, **His162**, and **Ala138**, and an amide–π stacked interaction with **Ala138**. A single unfavorable donor–donor interaction is observed with **Gly66**, highlighted in red. Image generated using BIOVIA Discovery Studio Visualizer v24.1.0.23298 [30].

Structural analysis of the cruzain—**H10** complex (Figure 4) reveals efficient accommodation of the ligand within the catalytic cleft, supported by a network of hydrophobic and π-interactions that explain its high binding affinity (IC_50_ = 0.512 µM).

**H10** adopted a stable orientation within the cruzain pocket, anchored by π–sulfur contacts with Met68/Trp26 and π–alkyl interactions with **Leu160**, **His162**, and **Ala138**. Minor donor–donor repulsion with **Gly66** was observed, but the overall hydrophobic network supports its potent enzymatic inhibition. Similarly, **H11** engaged π–sulfur (Met68) and π–alkyl (**Leu160/Asp161**) contacts, together with an amide–π interaction at **Ala138**, stabilizing the aromatic scaffold and accounting for its low-micromolar potency (IC_50_ = 0.412 µM).

The enzymatic potencies of **H7/H10/H11** (0.31–0.51 µM) fall within the micromolar range reported for TSCs and related scaffolds [6,25,32,33], although still below the nanomolar potencies achieved by covalent cruzain inhibitors. This positioning underscores the viability of the TSC scaffold as a starting point, suggesting further optimization to improve potency and selectivity.

### 3.3. Pharmacokinetic and Pharmacodynamic Predictions

The pharmacokinetic and pharmacodynamic evaluation, based on in silico tools such as SwissADME, Osiris, and pkCSM, showed that the majority of TSCs complied with Lipinski’s Rule of Five, indicating favorable oral bioavailability. Key parameters such as molecular weight (<500 Da), cLogP (<5), hydrogen bond donors (≤5), and hydrogen bond acceptors (≤10) were within optimal ranges for most compounds.

Topological Polar Surface Area (TPSA) values were also below 140 Å^2^ in nearly all derivatives, suggesting adequate membrane permeability. Compounds **H7**, **H10**, and **H11**, in particular, exhibited ideal physicochemical profiles with balanced lipophilicity and polarity—a combination strongly associated with both enzymatic potency and good cellular uptake.

Toxicity predictions flagged **H3** and **H15** due to the presence of nitro groups, which are often linked to mutagenic and genotoxic effects. This aligns with their observed cytotoxicity in vitro and reinforces the importance of avoiding such moieties in future designs. No significant hepatotoxicity or blood–brain barrier (BBB) permeability concerns were detected for the leading candidates, minimizing the risk of Central Nervous System (CNS) side effects.

Regarding metabolism and clearance, most compounds showed acceptable predicted hepatic clearance and low potential for cytochrome P450 inhibition. These parameters support the suitability of the most active compounds—particularly **H7**, **H10**, and **H11**—for further in vivo development, as they are unlikely to suffer from poor metabolic stability or rapid elimination.

### 3.4. Structure-Activity Relationship (SAR) Insights

Detailed SAR analysis of the top-performing cruzain inhibitors—**H7**, **H10**, and **H11** –highlights several critical molecular features that govern both binding affinity and biological performance. These compounds share a thiosemicarbazone scaffold extended by additional aromatic systems, which proved essential for effective engagement with the cruzain active site.

One of the most defining features of these derivatives is the **expanded aromatic surface area**, which facilitates multiple π–π stacking, π–sulfur, and π–alkyl interactions with hydrophobic residues, notably **Met68**, **Leu67**, **Leu160**, and **Trp26**. This expansion allows the ligands to span across both the S2 and S1′ subsites of the enzyme, maximizing contact and stabilization. In particular, the terminal aromatic rings in **H7** and **H11** align with lipophilic pockets and are further stabilized by favorable stacking against the side chains of methionine and tryptophan residues.

**Electron-donating substituents**, such as methoxy groups (e.g., in **H11**), not only enhance the electronic density of the aromatic system but also contribute to improved hydrogen bond acceptor ability, promoting interactions with polar residues like **Gly66** and **Asp161**. This electronic enrichment may also explain the increased hydrogen bonding and improved docking orientation seen in **H11**, even in the presence of some steric and electronic strain.

A key differentiating factor across the three leads is the **balance between favorable and unfavorable polar interactions**. **H7** stands out with the most favorable profile, forming strong hydrogen bonds with **Gly66**, **Gly163**, and **Cys25**, without significant donor–donor repulsion. In contrast, **H10** and **H11**, while still potent, present **unfavorable donor–donor interactions** involving **Gly66** and **Cys25**, potentially due to spatial congestion or improper orientation of hydrogen bond donors near polar residues. Although these do not eliminate binding, they may slightly reduce binding energy or introduce localized strain, suggesting a direction for refinement.

In terms of lipophilicity and polarity, **H7, H10**, and **H11** maintain an ideal balance. Their LogP values and topological polar surface areas (TPSA) remain within optimal ranges for oral bioavailability and passive membrane permeability. Importantly, none of the three compounds displayed predicted blood–brain barrier (BBB) penetration or hepatotoxicity, and all showed negligible in vitro cytotoxicity against human fibroblasts (CC_50_ > 64 µM), indicating a favorable safety window and minimal risk for CNS-related side effects.

Additionally, **amide–π stacking interactions** observed in **H10** and **H11** (particularly with **Ala138** and **Asp161**) highlight the relevance of maintaining a **planar conformation** near the thiosemicarbazone core, which appears to be well-tolerated within the enzyme’s relatively flat binding groove. Disruption of this planarity by bulky or non-coplanar substituents (as previously observed in less active compounds) generally leads to poor accommodation and reduced inhibitory activity.

Collectively, these findings emphasize that optimal cruzain inhibitors should:

- Maximize hydrophobic and π-interactions in the S2/S1′ subsites;
- Favor planar, conjugated systems for alignment with flat binding surfaces;
- Include electron-donating groups to support polar interactions without compromising solubility;
- Minimize steric hindrance near the hydrazone-thione region to avoid polar repulsion;
- Avoid strong electron-withdrawing or redox-a10/9/2025ctive groups such as nitro functionalities, as seen in **H3** and **H15**, which were linked to elevated cytotoxicity.

To contextualize the potency of our top hits (**H7, H10**, **H11**), we compared their enzymatic activity with previously reported cruzain inhibitors. While their IC_50_ values (0.31–0.51 µM) are comparable to early-generation scaffolds such as thiosemicarbazones and chalcone derivatives [8,25], they remain above the sub-micromolar to nanomolar potencies recently achieved by covalent cruzain inhibitors and optimized heterocycles [6,33]. This benchmarking underscores that our series constitutes a valuable starting point, with clear room for further structural optimization to approach the potencies of current preclinical candidates.

The performance of **H7**, **H10**, and **H11** underscores the success of this design strategy and provides a robust foundation for the rational development of next-generation cruzain inhibitors with optimized activity, selectivity, and safety profiles.

### 3.5. Mechanistic and Structural Insights into Cruzain Inhibition

**Figure 5:**
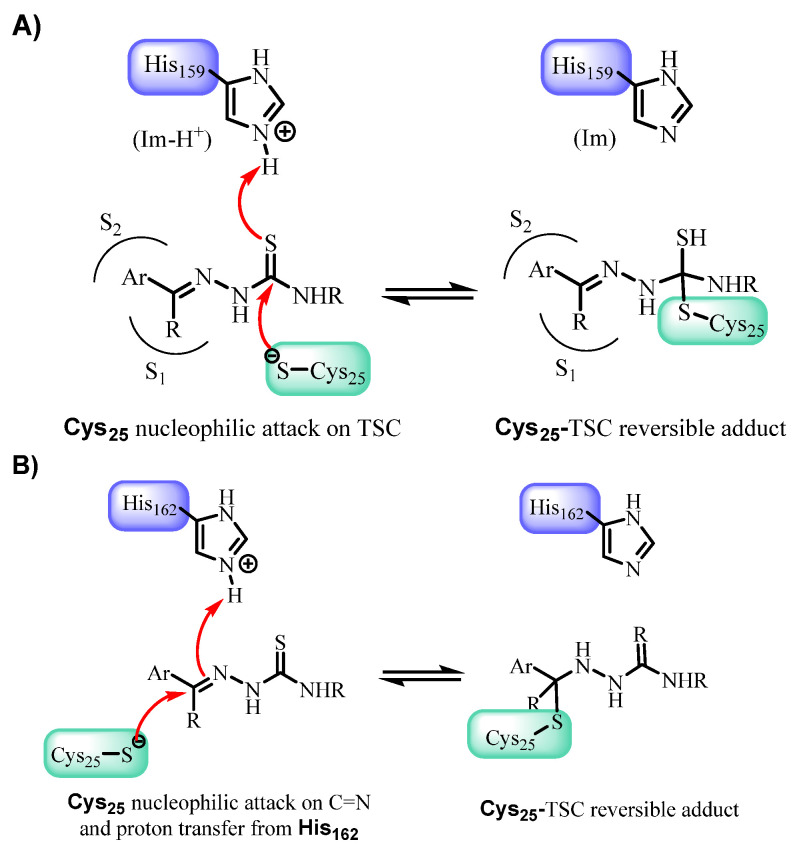
Proposed mechanisms of cruzain inhibition by TSCs. Adapted with permission from Ref. [34]. (**A**) Nucleophilic attack of the catalytic cysteine (Cys25) on the thiosemicarbazone (TSC), leading to the formation of a reversible Cys25–TSC adduct. (**B**) Nucleophilic attack of Cys25 on the C=N bond of the TSC, with concomitant proton transfer from His162, also resulting in a reversible Cys25–TSC adduct.

Two main mechanistic pathways have been proposed for cruzain inhibition by TSCs (Figure 5).

**Figure 6:**
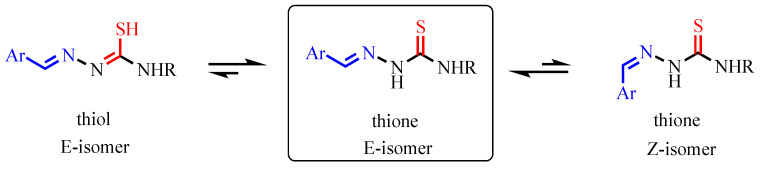
Illustration of the isomeric and tautomeric structures of TSCs. The different colors indicate specific atoms/groups: blue for the aromatic moiety (Ar), red for sulfur atoms, and black for carbon, nitrogen, and hydrogen atoms. The double-headed arrows represent tautomeric or isomeric equilibria between the different forms. The square in the middle highlights the thione E-isomer as the predominant form under polar conditions.

These two routes are not mutually exclusive and may be influenced by the electronic nature of the substituents and the tautomeric state of the ligand under physiological conditions. Conformational analysis and literature evidence suggest that under polar conditions—such as those mimicking the cruzain active site—TSCs predominantly exist in their *E*-isomeric form and as thione tautomers (Figure 6) [35]. This is significant because the thione form offers a better orientation and electron density for nucleophilic attack by the active-site cysteine, supporting the first mechanism as the most likely in this context.

Furthermore, the geometry of the *E*-isomer facilitates proper alignment within the enzyme pocket, minimizing steric clashes and optimizing interactions with the catalytic triad. This structural preference helps explain the higher potency observed in compounds like **H7**, **H10**, and **H11**, which combine favorable electronic features with optimal geometry for enzyme binding and inhibition.

Collectively, the integrated in silico and in vitro results validate the strategic value of targeting cruzain with rationally designed TSCs. The consistency across molecular docking, SAR insights, pharmacokinetic predictions, and biological assays supports the continued preclinical development of **H7**, **H10**, and **H11** as promising candidates for Chagas disease treatment.

## 4. Conclusions

This study successfully identifies thiosemicarbazone derivatives, particularly **H7**, **H10**, and **H11**, as highly promising cruzain-targeting trypanocidal agents for Chagas disease treatment. Through an integrated in silico and in vitro approach, these compounds demonstrated potent enzymatic inhibition, exceptional anti-*Trypanosoma cruzi* activity, and minimal cytotoxicity against human fibroblasts, resulting in high selectivity indices. Molecular docking revealed crucial interactions within the cruzain active site, supported by strong structure-activity relationships that emphasize the importance of electron-donating groups and expanded hydrophobic surfaces for optimal binding and specificity. Furthermore, ADMET predictions indicated favorable pharmacokinetic profiles and low toxicity risks for the lead compounds. The consistent correlation between computational predictions and experimental validations underscores the reliability of the multidisciplinary strategy employed. These findings not only highlight the therapeutic potential of these thiosemicarbazones but also provide a robust foundation for their further development as safe and effective anti-Chagas agents, reinforcing the value of rational drug design in addressing neglected tropical diseases.

As next steps, we propose in vivo evaluation (murine model of *T. cruzi* infection), solubility and metabolic stability assays (hepatic microsomes), CYP interaction profiling, and structure-guided optimization aimed at reducing polar repulsions in S2/S1′ and eliminating nitro-related toxicophores.

While the present study provides promising in silico and in vitro evidence supporting TSCs as cruzain-targeting anti-*T. cruzi* candidates, these results should be regarded as an early stage of drug discovery. Significant translational steps, including in vivo validation, pharmacokinetic optimization, and comprehensive safety evaluation, are still required before any potential clinical application can be envisioned.

Future work should aim to optimize the TSC scaffold by improving pharmacokinetic behavior, minimizing potential toxicophores, such as nitro groups, and enhancing selectivity. Overall, this research reinforces the value of integrating computational and experimental methods in the rational development of cruzain inhibitors and contributes meaningfully to drug discovery efforts targeting neglected tropical diseases.

## Figures and Tables

**Table 1:** Cruzain enzyme inhibition assays, phenotypic assays and cytotoxicities of TSCs.

Compound	Cruzain IC_50_ (µM)	*T. cruzi* CC_50_ (μM) ± SD	HFF-1 CC_50_ ± SD
**H1**	0.720 ± 0.015	09.80 ± 0.05	>64
**H2**	0.673 ± 0.065	10.15 ± 0.15	>64
**H3**	0.654 ± 0.085	04.23 ± 0.23	45.71 ± 1.03
**H4**	0.678 ± 0.026	08.60 ± 0.25	>64
**H5**	0.675 ± 0.010	12.05 ± 0.85	>64
**H6**	0.640 ± 0.028	07.65 ± 0.35	>64
**H7**	0.306 ± 0.009	01.96 ± 0.05	>64
**H8**	0.655 ± 0.015	08.15 ± 0.25	>64
**H9**	0.685 ± 0.045	10.15 ± 0.45	>64
**H10**	0.512 ± 0.012	02.85 ± 0.13	>64
**H11**	0.412 ± 0.042	02.15 ± 0.26	>64
**H12**	0.699 ± 0.024	09.25 ± 0.35	>64
**H13**	0.636 ± 0.026	10.68 ± 0.15	>64
**H14**	0.605 ± 0.015	10.95 ± 0.54	>64
**H15**	0.630 ± 0.041	05.87 ± 0.05	51.26 ± 0.87
**H16**	0.519 ± 0.023	2.95 ± 0.65	>64

## Data Availability

The original contributions presented in this study are included in the article. Further inquiries can be directed to A.S.d.O.
